# Feasibility and Acceptability of a Health App Platform Providing Individuals With a Budget to Purchase Preselected Apps to Work on Their Health and Well-Being: Quantitative Evaluation Study

**DOI:** 10.2196/51408

**Published:** 2024-05-29

**Authors:** Romy Fleur Willemsen, Niels Henrik Chavannes, Jiska Joëlle Aardoom

**Affiliations:** 1 Department of Public Health and Primary Care Leiden University Medical Center Leiden Netherlands; 2 National eHealth Living Lab Leiden University Medical Center Leiden Netherlands

**Keywords:** acceptability, accessible, adoption, application, design, ehealth, engagement, evaluation study, feasibility, health app platform, health apps, health empowerment, prevention, public health, uptake, user-friendly, users, wellbeing

## Abstract

**Background:**

The potential of health apps for health promotion and disease prevention is widely recognized. However, uptake is limited due to barriers individuals face in finding suitable and trustworthy apps, such as the overwhelming amount of available health apps. Therefore, the health app platform “FitKnip” was developed, enabling individuals to purchase preselected, trustworthy health apps with a budget of 100 euros (a currency exchange rate of EUR €1=US $1.0831 is applicable). The platform aimed to empower individuals to improve their health and vitality, ultimately supporting a more healthy society.

**Objective:**

The primary aim of this study was to evaluate the health app platform in terms of feasibility and acceptability. Potential effects on health empowerment and health outcomes were secondarily explored.

**Methods:**

This quantitative study was part of a mixed methods study with a prospective pre-post interventional design. We collected web-based user data, and self-reported web-based questionnaires were collected over 5 measurements over an 8-month period. Use statistics were tracked on the platform, including the number of purchased apps and euros spent per user registered within the health app platform. We measured the user-friendliness of the health app platform using the System Usability Scale (SUS) and satisfaction using the Client Satisfaction Questionnaire–8 (CSQ-8) and several 10-point Likert items. We asked participants to indicate, on a scale from 1 (not at all) to 10 (completely), how much the health app platform contributed to various areas related to health empowerment. We assessed health-related quality of life by the 12-item Short-Form Health Survey (SF-12) and one’s perceived level of stress by the 10-item Perceived Stress Scale (PSS-10).

**Results:**

A total of 1650 participants were included, of whom 42% (685/1650) bought at least 1 app. The majority of those purchased one app (244/685, 35.6%). The health app platform was rated as user-friendly (SUS mean 66.5, SD 20.7; range 66.5-70.0), and the acceptability of the health app platform was moderate (CSQ-8 mean 20.0, SD 1.5; range 19.6-20.0). Results furthermore showed that participants were generally satisfied to highly satisfied with the ease of the payment system to purchase apps on the platform (median 8, IQR 7-10), the look and feel of the platform (median 7, IQR 6-8), as well as the provided budget of 100 euros (median 9, IQR 7-10). Participants were less satisfied with the amount (median 6, IQR 4-7) and diversity (median 6, IQR 4-7) of apps offered on the platform.

**Conclusions:**

A health app platform is a promising initiative to enhance public health. Feasibility and acceptability are critical for success, as they ensure that such a platform is accessible, user-friendly, and meets end users’ needs and preferences. This can help to increase uptake, engagement, and ultimately the platform’s adoption and effectiveness.

## Introduction

The potential of health apps for health promotion and disease prevention is widely recognized [1]. They can provide individuals with tools to manage their well-being and disease, aid in self-diagnosis, provide medication reminders, and assist with rehabilitation [2]. Such apps can target a wide variety of health areas, such as nutrition, fitness, mindfulness, sleep, reproductive health, chronic diseases, substance abuse, depression, and anxiety [2,3]. Studies have shown that health apps can be effective in promoting healthy behaviors and self-management and can lead to increased health empowerment [4,5].

The number of health apps is on the rise, and an estimated total of 350,000 health apps were available for citizens in app stores in 2020 [6]. However, not all individuals find their way toward health apps due to barriers such as limited awareness about the availability of apps and low eHealth literacy [7,8]. Once individuals are aware of health apps, several other barriers may prevent them from downloading or using them, such as privacy concerns, a lack of scientific evidence on the effectiveness and efficacy of these apps, inadequate evaluation of quality, and unclarity concerning how the app is financed [2,3,9-13]. Concerning paid apps specifically, individuals are generally willing to pay for an app, but only if the app offers additional functionalities and features that are not available in free apps [7]. Moreover, due to the current health app overload, individuals are experiencing difficulties finding suitable and reliable apps [14].

To overcome the abovementioned barriers pertaining to the awareness, uptake, and use of health apps, a national experiment was initiated by the Dutch Ministry of Health, Welfare, and Sport. The experiment was also aimed at empowering individuals to work on their health and vitality, ultimately supporting a more healthy society. In this experiment, individuals from the general population were given access to a health app platform called “FitKnip.” The name FitKnip is based on a combination of the words “fit” and “knip,” referring to being physically and mentally fit and a wallet, respectively. On this health app platform, individuals were able to purchase preselected health apps with a personal digital health budget of 100 euros (a currency exchange rate of EUR €1=US $1.0831 is applicable). The preselection of apps ensured that the apps offered were reliable and trustworthy. Offered apps aligned with the concept of “positive health,” where the emphasis is not on illness but on people’s resilience in dealing with physical, emotional, and social challenges and on empowering individuals to take control of their health and well-being [15]. The primary aim of this study was to evaluate the health app platform in terms of feasibility and acceptability. Health empowerment and health outcomes while using the platform were explored.

## Methods

### Ethical Considerations

The study was declared to not fall within the scope of the Dutch Medical Research Involving Human Subjects Act by the Medical Ethics Committee Leiden-Den Haag-Delft (N19.0878) as the study was non-invasive. Participants signed a digital informed consent form before participating. To ensure privacy, the data set displayed only participant numbers (ie, pseudonymized data), and the combination of participant numbers and email addresses was securely stored in a separate protected file. Participants were not reimbursed for completing the questionnaires.

### Study Design

**Figure 1:**
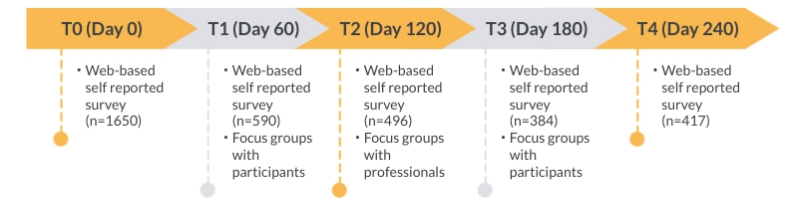
Overview of the mixed methods study.

The current quantitative study is part of a mixed methods study with a prospective pre-post interventional design (Figure 1). The qualitative results (ie, focus group interviews) are reported elsewhere [16]. This study used web-based user data and questionnaires as obtained over 5 measurements over a period of 8 months: T0 (baseline), T1 (60 days), T2 (120 days), T3 (180 days), and T4 (240 days) after baseline, respectively. The study was declared to not fall within the scope of the Dutch Medical Research Involving Human Subjects Act by the Medical Ethics Committee Leiden-Den Haag-Delft.

### Population and Recruitment

A total of 2562 participants were recruited on the web and offline through social media posts, newsletters, advertisements in local newspapers, and personal communication by various institutions in the Netherlands. These institutions included health insurance companies, a health care coalition, an academic hospital, the Ministry of Health, Welfare, and Sport, an employers’ organization, local governments, municipality teams, a knowledge and quality institute for oncological and palliative care, as well as a web-based platform for patients with cancer and their caregivers. Interested individuals could submit their email addresses, and they were subsequently invited to participate in the study. The invitation email included a web-based information letter about the study and a link to the research environment, where individuals were screened for eligibility through self-report questions. The inclusion criteria were as follows: (1) being aged 18 years or older; (2) being able to understand, read, and speak the Dutch language; and (3) having access to the internet. If eligible, individuals were asked to provide digital informed consent and to complete the web-based baseline questionnaire (T0). Hereafter, individuals were provided access to the health app platform.

### Intervention

Participants received access to the FitKnip health app platform, which enabled them to purchase preselected, trustworthy apps. Apps were preselected by a health care coalition based on the following criteria: (1) the app must be ready for use and can be used without the involvement of a health care professional; (2) the app should not include in-app purchases; (3) the provider of the app must adhere to national and international laws, such as the General Data Protection Regulation; and (4) the app should enable anonymous purchases, and the provider of the app should not be allowed to publish or sell user data.

A total digital budget of 100 euros was available for each participant. The budget could be used during the entire research period of 8 months. The platform offered a total of 38 apps, which could be categorized into 1 or more of the six dimensions of positive health [15], namely, (1) bodily functions, (2) mental functions and perception, (3) spiritual or existential dimensions, (4) quality of life, (5) social and societal participation, and (6) daily functioning. For example, apps can help users live more healthily, sleep better, process grief, or communicate more clearly.

The majority of these apps were progressive web applications (PWAs). PWAs are web apps that offer mobile app–like experiences while giving the user a faster and more reliable version of the app. These PWAs are, from here on, referred to as apps in this manuscript.

### Outcomes

#### Sociodemographic and Clinical Characteristics

All sociodemographic and clinical characteristics were assessed at T0. Sociodemographic characteristics included age, gender, country of birth, educational level, work status, living status, and BMI. Clinical characteristics were assessed by asking whether the individual was diagnosed with any mental or medical condition (“yes” or “no”) and, if so, the type of diagnosis and the severity of symptoms (on a scale of 1 to 10). Other questions were related to the frequency of health care use during the past month.

We assessed eHealth literacy using the eHealth Literacy Questionnaire (eHLQ) [17], which is a validated multidimensional tool based on the eHealth Literacy Framework. The questionnaire includes 35 statements, and individuals are asked to indicate to what extent they agree with a certain statement on a 4-point Likert scale ranging from “strongly disagree” to “strongly agree.” The questionnaire comprises 7 dimensions, such as “using technology to process health information.” Scores per dimension are calculated by averaging the item’s scores within each scale with equal weighting, which generates scale scores that range from 1 to 4. Information about the use of the eHLQ in the Dutch context can be found elsewhere [18].

#### Feasibility

Use statistics, more specifically the number of purchased apps and the amount of budget spent in euros per user, were registered within the health app platform. Moreover, at baseline, participants were asked for their reasons for participating in the experiment. Participants could select 1 or more predetermined answer categories (eg, improving general, mental, or physical health). They were also asked which types of apps they were interested in, as assessed based on the 6 dimensions of positive health.

#### Acceptability

All acceptability questionnaires were administered at T1, T2, T3, and T4.

The user-friendliness of the health app platform was measured by the System Usability Scale (SUS) [19]. The SUS is a robust, valid, and versatile questionnaire to help assess the user-friendliness of a system or product [20]. The questionnaire consists of ten 5-point Likert items, with answer categories ranging from “totally disagree” to “totally agree.” The total score (ranging from 0 to 100) is the sum of the individual item scores. The higher the score, the higher the user-friendliness. A website is considered to be above-average in user-friendliness when the SUS score is above 68 [19].

Satisfaction with the health app platform was measured with the Client Satisfaction Questionnaire–8 (CSQ-8) [21,22]. The questionnaire consists of 8 multiple-choice questions with 4 corresponding answer categories each. Participants are, among others, asked about their satisfaction with the health app platform, whether the platform has met their needs and preferences, and whether they would recommend the platform. The total score ranges from 8 to 32, with higher scores reflecting higher satisfaction. The questionnaire has good psychometric characteristics (Larsen et al [22]).

The satisfaction with the available type and number of apps on the platform, the look and feel of the platform, as well as the provided budget and the ease of the payment system, was evaluated on a 10-point Likert scale ranging from 1 (very dissatisfied) to 10 (very satisfied).

#### Health Empowerment and Health Outcomes

All health empowerment questions were administered at T1, T2, T3, and T4. Participants were asked to indicate, on a scale from 1 (not at all) to 10 (completely), how much the health app platform contributed to improvement in various areas related to health empowerment: health and vitality, health awareness, perceived control in regard to one’s health and vitality, receiving appropriate help, and helping to deal more effectively with health problems encountered. These questions were formulated by the research team. Items were answered on a scale from 1 (very dissatisfied) to 10 (very satisfied), with a nonapplicable option where appropriate.

Health-related quality of life was assessed by the 12-Item Short-Form Health Survey (SF-12) [23]. The SF-12 measures physical and mental health by means of 2 summary scores: the physical component summary (PCS) and the mental component summary (MCS). The PCS is comprised of physical functioning (2 items), role limitations due to physical health problems (2 items), bodily pain (1 item), and general health (1 item). The MCS is comprised of vitality and energy (1 item), social functioning (1 item), role limitations due to emotional problems (2 items), and mental health and psychological well-being (2 items). The scoring was conducted using the SF-12 scoring manual [23,24]. Scores could range from 1 to 100 for both the PCS and MCS. Higher scores indicate a better general health status. The norm score for the general population is 50 [25].

One’s perceived level of stress was determined by the 10-item Perceived Stress Scale (PSS-10) [26]. The PSS-10 is a widely used measure of global perceived stress and comprises 2 underlying factors: perceived helplessness and perceived self-efficacy [27]. Example questions are “Have you been upset because of something that happened unexpectedly?” and “Have you felt that things were going your way?” Questions are answered on a scale from 0 (never) to 4 (often). A total score was calculated (ranging from 0 to 40) and can be divided into low (0-13), moderate (14-26), or high stress (27-40). The PSS-10 is found to be a robust predictor of health and disease [28,29].

### Data Analysis

All quantitative analyses were performed in SPSS (version 24.0; IBM Corp). Descriptive analyses, that is, mean (SD), median (IQR), and n (%), were used to describe the sociodemographic and clinical characteristics of the study population, as well as study outcomes. Health outcomes were evaluated for changes over time. Therefore, the health outcomes on T1-T4 were one-by-one statistically compared to T0 using linear mixed model analyses. These models included a random intercept to account for within-subject correlation among repeated measures, as through the –2 restricted likelihood test it was determined that including random slopes led to a better model fit. Time contrasts were created using dummy coding (T0=1; T1=2; T2=3; and T4=3). Age and gender were forced into the models as covariates. Next to this, a stepwise forward selection approach was used to select potential confounders and covariates in the associations between time and health outcomes, more specifically education and work status. However, the addition of both education and work status did not lead to a change of >10% in β coefficient; hence, they were not included in the models. Furthermore, we evaluated whether change over time for health outcomes differed between subgroups (ie, effect modification) based on age categories, gender, and diagnosis. The variable diagnosis was an effect modifier in the SF-12 PCS and MCS scores and the PSS-10 scores. Therefore, the results for these outcomes were reported separately for subgroups of participants with and without a diagnosis.

## Results

### Sociodemographic and Clinical Characteristics

The sociodemographic and clinical characteristics are shown in Table 1. The average age of the study population was approximately 45 (range 18-81) years. The majority were female (n=1177, 71.3%), highly educated (n=1307, 79.2%), had a full-time (n=722, 43.8%) or part-time (n=450, 27.2%) job, and was living together with a partner (n=545, 33%) or with both a partner and children (n=580, 35.2%). Approximately half of the study population had a healthy BMI, whereas roughly 30% were overweight and 18% were obese.

**Table 1:** Sociodemographic and clinical characteristics of the study population (N=1650).

Characteristics		Frequency
Age (years), mean (SD)		45.1 (13.3)
**Age (years), n (%)**		
	18-29	230 (13.9)
	30-39	404 (24.5)
	40-49	364 (22.1)
	50-64	525 (31.8)
	≥65	127 (7.7)
**Gender, n (%)**		
	Male	467 (28.3)
	Female	1177 (71.3)
	Neutral	6 (0.4)
**Country of birth, n (%)**		
	Netherlands	1556 (94.3)
	Germany	10 (0.6)
	Suriname	16 (0.1)
	Other^a^	68 (4.1)
**Education, n (%)**		
	Low	58 (3.5)
	Middle	285 (17.3)
	High	1307 (79.2)
**Work status, n (%)**		
	Student	58 (3.6)
	Full-time job	722 (43.8)
	Part-time job	450 (27.2)
	Volunteer	45 (2.7)
	Retired	111 (6.7)
	Incapacitated	113 (6.8)
	Sick leave	60 (3.6)
	Other	91 (5.5)
**Living situation, n (%)**		
	Living with partner	545 (33.0)
	Living with partner and children	580 (35.2)
	Student housing or with friends	45 (2.7)
	Alone	316 (19.2)
	Other	164 (9.9)
BMI (kg/m^2^), mean (SD)		25.8 (5.2)
**BMI (kg/m^2^), n (%)**		
	<18.5 (underweight)	33 (2.0)
	18.5-24.99 (healthy weight)	818 (49.6)
	25-29.99 (overweight)	498 (30.2)
	>30 (obese)	299 (18.1)
	Missing	2 (0.1)
**Medical, physical, or psychological diagnosis, n (%)**		
	Yes	592 (35.9)
	No	1058 (64.1)
Severity of symptoms (scale of 1-10), mean (SD)		5.8 (2.2)
**Health care use past month, n (%)**		
	Yes	635 (38.5)
	No	1015 (61.5)
**Type of health care (in case of health care use)**		
	General practitioner	310 (18.8)
	Medical specialist	276 (16.7)
	Physiotherapist	168 (10.2)
	Dietician	31 (1.9)
	Psychology	152 (9.2)
	Other	124 (7.5)
**Frequency of health care use (past month), n (%)**		
	Once or a couple of times (<1 per week)	466 (28.2)
	Occasionally (once a week)	116 (7.0)
	On a regular basis (several times per week)	49 (3.0)
	Often (most days of the week)	4 (0.2)
	Total	635 (38.4)

^a^Angola, Aruba, Belgium, Bolivia, Brazil, Bulgaria, Columbia, Curaçao, Czech Republic, Finland, France, Hungary, Indonesia, Iran, Ireland, Israel, Italia, Kenia, Lithuania, Macedonia, Morocco, Mexico, Norway, Peru, Philippines, Russia, Saudi Arabia, Senegal, Sweden, Swiss, Syria, Turkey, United Kingdom, United States, Venezuela, Zambia, and Zimbabwe.

Approximately one-third (592/1650, 35.9%) of the study population reported having a medical, physical, or psychological diagnosis, and 38.5% (635/1650) of the participants reported health care use in the past month, of whom most went to a general practitioner (310/635, 52.4%) or a medical specialist (276/635, 46.6%).

Most participants scored on or above the middle of the scale on the eHLQ. These results suggest sufficient-to-good eHealth literacy skills (Table S1 in Multimedia Appendix 1).

### Feasibility

Platform use data showed that 42% (685/1650) of participants purchased at least 1 app (Table 2). Of those, the majority of participants purchased 1 app (244/685, 35.6%), followed by 2 (175/685, 25.5%) and 3 apps (104/685, 15.2%). Only a minority of participants (18/685, 2.5%) purchased over 10 apps. At T1, the majority of participants purchased 1 app (291/685, 42.5%), whereas at T2, T3, and T4, most participants did not purchase any apps (512/685, 74.7%; 623/685, 90.9%; and 601/685, 87.7%, respectively). Detailed results on the budget spent per end user can be found in Table 2. The top 5 apps purchased by participants were as follows: (1) an activity tracker (173 times), (2) a mindfulness app (171 times), (3) a healthy living app (142 times), (4) a communication app providing insight and exercises in personal communication styles (121 times), and (5) an app targeting self-image (109 times).

**Table 2:** Objective use statistics of the health app platform (n=685).

		T1	T2		T3		T4	Total
Number of purchased apps per end user, median (IQR)		1.00 (1.00-2.00)	0.00 (0.00-1.00)		0.00 (0.00-0.00)		0.00 (0.00-0.00)	2.00 (1.00-3.00)
**Number of purchased apps per end user, n (%)**								
	0	91 (13.3)		512 (74.7)		623 (90.9)	601 (87.7)	N/A^a^
	1	291 (42.5)		104 (15.2)		42 (6.1)	42 (6.1)	244 (35.6)
	2	152 (22.2)		40 (5.8)		11 (1.6)	16 (2.3)	175 (25.5)
	3	73(10.7)		11 (1.6)		6 (0.9)	4 (0.6)	104 (15.2)
	4	29 (4.2)		4 (0.6)		1 (0.1)	1 (0.1)	49 (7.2)
	5	14 (2.0)		5 (0.7)		0 (0.0)	3 (0.4)	5 (5.1)
	6-10	32 (4.7)		8 (1.2)		2 (0.2)	13 (1.90)	60 (8.7)
	>10	3 (0.4)		1 (0.1)		0 (0.0)	5 (0.73)	18 (2.5)
Euros spent per end user, mean (SD)		22.12 (5.00-30.23)	5.01 (0.00-5.00)		0.00 (0.00-0.00)		0.00 (0.00-0.00)	24.00 (9.5-57.00)
**Euros^a^ spent per end user**								
	0, n (%)	0 (0)		512 (74.4)		630 (92)	604 (88.3)	—^b^
	1-25, n (%)	496 (72.4)		135 (19.7)		47 (6.9)	50 (7.3)	356 (52.0)
	26-50, n (%)	45 (6.6)		19 (2.8)		6 (0.9)	16 (2.3)	128 (18.7)
	51-75, n (%)	21 (3.1)		14 (2.0)		2 (0.3)	5 (0.7)	132 (19.3)
	76-100, n (%)	23 (3.4)		4 (0.6)		0 (0.0)	9 (1.3)	68 (9.9)
	Total, n (%)	685 (100)		684 (100)		685 (100)	684 (100)	684 (100)
	Missing, n	0		1		0	1	1

^a^A currency exchange rate of EUR €1=US $1.0831 is applicable.

^b^Not applicable as this table shows the results of the subgroup of participants who bought at least 1 app.

The 3 most selected reasons for participants to try out the health app platform were to improve general health (989/1650, 59.9%), be interested in health (913/1650, 55.3%), and improve physical health (696/1650, 42.2%) (Table S2 in Multimedia Appendix 1). The most selected app interest areas among participants were “bodily functions” (1335/1650, 80.9%) and “mental functions and perceptions” (938/1650, 56.8%); the least selected interest areas were “spiritual dimension” (512/1650, 31%) and “social and societal participation” (368/1650, 22.3%).

### Acceptability

Table 3 presents the results concerning the acceptability of the health platform. In general, user friendliness, as assessed by the SUS, was rated above average. The acceptability of the health app platform, as assessed by the CSQ-8, was scored as moderate. Furthermore, results showed that on average, participants were “satisfied” to “highly satisfied” with the ease of the payment system, the look and feel of the website, and the provided budget of 100 euros. Regarding the provided budget, approximately 40% (660/1650) of participants rated it a 10 on a scale of 1 to 10 (range T1-T4 38.5%-39.4%). Participants were less satisfied with the amount and type of apps. Around 40% (660/1650) scored these elements as insufficient (≤5), respectively, ranging from 39.8% to 47.2% and from 41.1% to 45.1%.

**Table 3:** Acceptability and health empowerment of the health app platform.

		T1 (n=579)	T2 (n=360)	T3 (n=285)	T4 (n=337)
**Acceptability, mean (SD)**					
	Client Satisfaction Questionnaire–8	20.0 (1.5)	19.7 (1.44)	19.6 (1.5)	19.6 (1.4)
	System Usability Scale	66.5 (20.7)	69.6 (18.0)	70.0 (18.4)	69.9 (19.7)
**Acceptability, median (IQR)**					
	Ease of payment system	8 (7-10)	8 (7-10)	8 (7-10)	8 (7-10)
	Satisfaction with the look and feel of health app platform	7 (6-8)	7 (6-8)	7 (6-8)	7 (6-8)
	Satisfaction with the amount of apps	6 (4-7)	6 (4-7)	6 (4-7.5)	6 (4-8)
	Satisfaction with type of apps	6 (4-7)	6 (4-7)	6 (4-7)	6 (4-7)
	Satisfaction with budget	9 (7-10)	9 (8-10)	9 (8-10)	9 (8-10)
**Health empowerment, median (IQR)**					
	Support in health and vitality	3 (1-6)	4 (2-6)	4 (2-6)	4 (2-6)
	Health awareness	5 (2-6)	5 (2-7)	5 (2-7)	5 (2-7)
	Control in regard to their health and vitality	4 (1-6)	4 (2-7)	4 (2-7)	5 (2-7)
	Receiving help or appropriate care	2 (1-5)	3 (1-5)	3 (1-6)	2 (1-6)
	Helping to deal more effectively with health problems encountered	3 (1-5)	3 (1-6)	3 (1-6)	4 (1-6)

### Health Empowerment and Health Outcomes

Concerning health empowerment, in general, participants did not perceive the health app platform as “supporting” (Table 3), especially in terms of the health app platform supporting them in finding appropriate help (range 2-3).

Both the physical and mental subscale scores of the SF-12 were stable across the research period, with scores below the norm of 50 for the general population (Table 4).

**Table 4:** Health outcomes over time.

	Measure			T0 (n=1650)		T1 (n=590)			T2 (n=496)			T3 (n=384)	T4 (n=417)	
**Short-Form Health Survey, mean (SD)**														
	**Mental component summary**													
		Total study population	45.9 (10.1)		47.3 (10.1)			46.4 (10.8)			47.2 (10.1)		46.1 (11.2)	
		Diagnosis (n=592)	43.9 (11.1)		45.0 (11.0)			45.0 (11.7)			45.3 (11.0)		44.1 (12.2)	
		No diagnosis (n=1058)	47.0 (9.4)		48.7 (9.1)			47.2 (10.1)			48.4 (9.3)		47.4 (10.1)	
	**Physical component score**													
		Total study population	47.2 (11.0)		46.1 (11.7)			46.6 (11.3)			47.1 (11.4)		47.1 (11.4)	
		Diagnosis (n=592)	40.8 (11.9)		39.0 (12.7)			40.0 (11.7)			40.9 (12.1)		41.5 (12.3)	
		No diagnosis (n=1058)	50.8 (8.5)		50.6 (8.4)			50.8 (8.7)			51.0 (8.9)		51.3 (8.7)	
**10-item Perceived Stress Scale, mean (SD)**														
	Total study population			15.1 (6.5)		14.2 (6.4)			14.3 (6.7)			14.0 (6.6)	14.3 (6.8)	
	Diagnosis (n=592)			17.1 (6.8)		16.4 (6.5)			16.0 (6.8)			15.8 (6.9)	16.0 (7.2)	
	No diagnosis (n=1058)			14.0 (6.1)		12.8 (6.0)			13.2 (6.4)			12.9 (6.2)	13.1 (6.3)	
Low (0-13), n (%)				700 (42.4)			284 (48.1)			237 (47.8)		191 (49.7)		206 (49.4)
Moderate (14-26), n (%)				867 (52.5)			284 (48.1)			237 (47.8)		179 (46.6)		189 (45.3)
High (27-40), n (%)				85 (5.1)			22 (3.7)			22 (4.4)		14 (3.6)		22 (5.3)

The subgroup analysis based on diagnosis showed that the mean MCS score for participants without a diagnosis was significantly higher on T1 (48.7, SD 9.1) compared to T0 (47.0, SD 9.4; β=1.15, SE 0.36; *P*<.001; Table S3 in Multimedia Appendix 1).

The PCS score on T1 was significantly lower, and thus declined, for participants with a diagnosis (β=–1.60, SE 0.74; *P*=.03). The PCS score for participants without a diagnosis was higher, and thus improved, on T4 (β=0.98, SE 0.44; *P*=.03; Table S4 in Multimedia Appendix 1).

Table 4 shows that participants’ perceived stress levels remained quite stable across the research period, indicating low to moderate levels of stress.

The subgroup analyses based on diagnosis showed that PSS-10 scores were significantly lower, and thus improved, for participants without a diagnosis on T1 (mean 12.8, SD 6.0; β=–0.91, SE 0.22; *P*<.001), T3 (β=–0.60, SE 0.29; *P*=.04), and T4 (β=–0.82, SE 0.29; *P*<.001) compared to T0 (mean 14.0, SD 6.1; Table S5 in Multimedia Appendix 1).

## Discussion

### Principal Results

This study aimed to evaluate the feasibility and acceptability of a health app platform where individuals were given a budget of 100 euros to purchase preselected, trustworthy health apps (primary aim), and to explore the potential impact on health empowerment and health outcomes (secondary aim). Results showed that of the 1650 participants, only approximately 42% (685/1650) purchased at least 1 app during the 8-month-long experiment. The number of app purchases among these participants was rather low, and the majority of participants had budgets left to purchase additional apps. In general, participants were satisfied with the health app platform and found the platform easy to use; participants were satisfied with the look and feel of the platform, the ease of the payment system, and the provided monetary budget. However, they were less satisfied with the number and diversity of apps offered on the platform. Results further suggested that the health app platform in its current form did not seem to contribute to participants’ health empowerment and outcomes.

### Comparison With Previous Work

Although during the current experiment, the user engagement stayed behind what was expected, the results of the qualitative study about this experiment showed that participants were enthusiastic about the concept of a health app platform to promote health and vitality. Furthermore, a previous study showed the added value of a health app platform called Intellicare with regard to the number of individual health app downloads [30]. Intellicare is a hub app, which is a catalog for individual apps targeting depression and anxiety [30,31]. The results of Lattie et al [30] showed that the number of individual health app downloads among hub app users was higher as compared to nonhub app users. Thus, while user engagement with the FitKnip platform was not as expected, the concept and function of a health app platform have shown potential in other studies.

A health app platform is meant to guide individuals to suitable health apps; however, in current initiatives, this process is not sufficiently supported [32,33]. Facilitating the selection process for individuals might be the key to higher app downloads.

Other factors that increase app downloads and user engagement on health app platforms are the incorporation of personalized reminders and personal contact or coaching [30-32]. Indeed, the results of a follow-up study of Intellicare suggest that personal coaching through a phone call and SMS text messages, aimed at encouraging engagement with the hub app and individual health apps, can indeed be an effective strategy for increasing app downloads [31]. Participants in the qualitative study also suggested incorporating contact with professionals into future health app platforms [32].

Studies of individual apps also offer ideas on how to increase engagement on a health app platform [34-37].

During the experiment, the study dropout increased over time; only approximately 1 in 4 participants completed the 8-month follow-up questionnaire. Such high study dropout rates are common in digital health studies [38-42]. Our results showed that participants with a medical, physical, or psychological diagnosis were less likely to drop out of the study. This is in line with previous literature, where low dropout levels were observed for individuals who perceive their own health to be poor [43], as well as those who want to be involved in their health care and who are interested in self-monitoring their health status [44]. These studies conclude that individuals with poor health, a chronic disease, or a family history of disease are more willing to work on their own health care [43,45,46]. More generally, by understanding different types of users and their motivations for using health apps, developers can create platforms that are better tailored to meet the needs of end users, or they can even create separate platforms for specific target groups and improve engagement accordingly [32,44].

The results indicated that, in general, the current health app platform did not meet participants’ needs in terms of supporting health empowerment, that is, in gaining the knowledge, skills, and confidence needed to take action to improve their health. This is in line with the results of this qualitative study, which showed that some participants believed that a certain level of health empowerment is a prerequisite for the use of a health app platform and that the health platform first needs to be improved to fulfill its potential [32]. Additionally, no major changes were observed in health outcomes over time. The platform should first be improved to better support individuals in their health in terms of the ability to select apps, the app catalog (eg, more physical health–related apps), and personalization in terms of language and type of apps offered [32]. More research is needed to gather more in-depth information about the barriers and facilitators to health empowerment and health outcomes, and how a health app platform could best support users in this process.

### Strengths and Limitations

To our knowledge, this is the first national, government-initiated experiment that aims to tackle barriers to the uptake of health apps by providing participants access to a platform of preselected, trustworthy health apps and providing them with a monetary budget to purchase these apps. The platform is therefore an innovative way to provide individuals with the tools to work on their own health and vitality. A strength of this study is the substantial study population of 1650 participants, as well as the evaluation of FitKnip in a real-world setting, providing a rather realistic representation of how users engage with the platform in their everyday lives.

Some study limitations should be considered when interpreting the findings, one of which is the generalizability of the study results. The current study population was predominantly female, highly educated, aged 30 years or older, and diagnosed with a medical, physical, or psychological issue. The health app platform might have been more useful and interesting for participants with a diagnosis, as they might have been more willing to work on their own health care, which could have positively biased results on the feasibility and acceptability of the platform. Furthermore, as young individuals (aged between 18 and 30 years) were underrepresented in the study population, the current results may not be generalizable to a younger population.

### Implications for the Future

The current results highlight the need for future health app platforms to place a strong emphasis on maximizing uptake and user engagement with the platform. This could be achieved by prioritizing the needs and perspectives of end users in the design and development of health app platforms and developing such a platform in cocreation to ensure that it is a valuable, empowering, and effective tool in promoting health and well-being. Cocreation is identified as essential for the success of eHealth initiatives [11,47]. Thus, it is crucial to involve end users already in the early stages of the development of an app or digital intervention through cocreation to increase engagement, adherence, and ultimately adoption.

### Conclusion

A shift from reactive to proactive care is a crucial step in improving health outcomes and reducing the burden on the health care system. A health app platform can support such proactive care by enabling and empowering individuals to work on their health, vitality, and well-being. Hence, a health app platform presents a promising opportunity to enhance public health. To ensure its success, however, feasibility and acceptability are paramount, as these aspects ensure the platform’s accessibility, user-friendliness, and alignment with the needs and preferences of end users. Addressing these factors is instrumental in boosting uptake and engagement, and ultimately, the platform’s adoption and effectiveness.

## Appendix

Multimedia Appendix 1 Supplemental tables on eHealth literacy, feasibility, and the linear mixed models for health outcomes.

## Abbreviations

CSQ-8: Client Satisfaction Questionnaire–8
eHLQ: eHealth Literacy Questionnaire
MCS: mental component summary
PCS: physical component summary
PSS-10: 10-item Perceived Stress Scale
PWA: progressive web application
SF-12: 12-item Short-Form Health Survey
SUS: System Usability Scale
WHO: World Health Organization

## Data Availability

The data sets generated or analyzed during this study are not publicly available since participants were not asked for consent to make their data publicly available, but are available from the corresponding author upon reasonable request.
