# Supplementary material for: Feasibility and Acceptability of a Health App Platform Providing Individuals With a Budget to Purchase Preselected Apps to Work on Their Health and Well-Being: Quantitative Evaluation Study
Source: JMIR Form Res. 2024 May 29;8:e51408. doi: 10.2196/51408 (PMC11170047; doi:10.2196/51408)
Supplement: Multimedia Appendix 1 [file formative_v8i1e51408_app1.docx]

*Table 1 eHealth literacy*

| Components of the eHealth Literacy Questionnaire (eHLQ) | M (SD)  (n=1650) |
| --- | --- |
| 1. Using technology to process health information | 2.8 (0.4) |
| 2. Understanding of health concepts and language | 3.0 (0.4) |
| 3. Ability to actively engage with digital services | 3.2 (0.5) |
| 4. Feel safe and in control | 2.8 (0.5) |
| 5. Motivated to engage with digital services | 2.9 (0.4 |
| 6. Access to digital services that work | 2.5 (0.5) |
| 7. Digital services that suit individual needs | 2.5 (0.5) |

*Table 2 Feasibility: Reasons to participate in FitKnip and interest areas apps at baseline*

|  | N | % |
| --- | --- | --- |
| Reasons to participate in FitKnip^1^  Improve my general health  Improve my physical health  Improve my mental health  Interest in health  Recommended by healthcare professional  Improve specific symptoms | 989  696  476  913  9  151 | 59.9  42.2  28.8  55.3  0.5  9.2 |
| Interest areas apps^1^  Spiritual dimension  Mental functions & perceptions  Quality of life  Daily functioning  Bodily functions  Social and societal participation  Do not know yet | 512  938  735  811  1335  368  316 | 31.0  56.8  44.5  49.2  80.9  22.3  19.2 |

^1^Participants could select multiple answer options

*Table 3 Linear mixed model of the Mental Component Score (MSC) of the 12-item Short Form health survey (SF-12) with time, gender and age. Separately reported for diagnosis.*

| Effect | Estimate (SE) | p-value |
| --- | --- | --- |
| **SF12 MCS with diagnosis** |  |  |
| Intercept | 34.81 (1.34 ) | <.01 |
| Time  *T0*  *T1 T2 T3 T4* | Ref  1.01 (0.77)  0.73 (0.82)  1.00 (0.91)  -0.11 (0.86) | 0.19  0.37  0.27  0.89 |
| Gender  *Male*  *Female*  *Neutral* | Ref  0.06 (0.63)  0.87 (4.51) | 0.92 0.85 |
| *Age* | 0.19 (0.02) | <.01 |
| **SF12 MCS without diagnosis** |  |  |
| Intercept | 41.67 (1.09) | <.01 |
| Time  *T0*  *T1 T2 T3 T4* | Ref  1.15 (0.36)  -0.69 (0.46)  0.21 (0.42)  -0.33 (0.45) | <.01  0.13  0.62  0.46 |
| Gender  *Male*  *Female*  *Neutral* | Ref  -1.22 (0.59)  2.02 (4.51) | 0.04  0.65 |
| *Age* | 0.14 (0.02) | <.01 |

Table 4 Linear mixed model of the Physical Component Score (PCS) of the 12-item Short Form health survey (SF-12) with time, gender and age. Separately reported for diagnosis.

| Effect | Estimate (SE) | P-value |
| --- | --- | --- |
| **SF12 PCS with diagnosis** |  |  |
| Intercept | 54.73 (1.28) | <.01 |
| Time  T0  T1 T2 T3 T4 | Ref  -1.60 (0.74)  -0.28 (0.79)  0.48 (0.87)  0.99 (0.82) | 0.03  0.73  0.58  0.23 |
| Gender  Male  Female  Neutral | Ref  3.54 (0.61)  6.48 (4.29) | <.01 0.13 |
| Age | -0.23 | <.01 |
| **SF12 PCS without diagnosis** |  |  |
| Intercept | 56.84 (0.98) | <.01 |
| Time  T0  T1 T2 T3 T4 | Ref  0.10 (0.31)  0.23 (0.39)  0.45 (0.44)  0.98 (0.44) | 0.75  0.56  0.31  0.03 |
| Gender  *Male*  *Female*  *Neutral* | Ref  -0.56 (0.53)  1.22 (4.09) | 0.30  0.76 |
| *Age* | -0.13 (0.02) | <.01 |

*Table 5 Linear mixed model of 10-item Perceived Stress Score (PSS-10) with time, gender and age. Separately reported for diagnosis.*

| Effect | Estimate (SE) | P-value |
| --- | --- | --- |
|  |  |  |
| **PSS-10 with diagnosis** |  |  |
| Intercept | 19.59 (0.87) | <.01 |
| Time  *T0*  *T1 T2 T3 T4* | Ref  -0.75 (0.50)  -0.98 (0.53)  -1.10 (0.59)  -0.87 (0.55) | 0.14  0.07  0.06  0.12 |
| Gender  *Male*  *Female*  *Neutral* | Ref  1.63 (0.41)  -4.03 (2.92) | <.01  0.17 |
| *Age* | -0.08 (0.01) | <.01 |
| **PSS-10 without diagnosis** |  |  |
| Intercept | 15.54 (0.71) | <.01 |
| Time  *T0*  *T1 T2 T3 T4* | Ref  -0.91 (0.22)  -0.36 (0.27)  -0.60 (0.29)  -0.82 (0.29) | <.01  0.18  0.04  <.01 |
| Gender  *Male*  *Female*  *Neutral* | Ref  1.68 (0.39)  -2.04 (2.95) | <.01  0.49 |
| *Age* | -0.06 (0.01) | <.01 |
